# Multi-voxel pattern analysis (MVPA) reveals abnormal fMRI activity in both the “core” and “extended” face network in congenital prosopagnosia

**DOI:** 10.3389/fnhum.2014.00925

**Published:** 2014-11-13

**Authors:** Davide Rivolta, Alexandra Woolgar, Romina Palermo, Marina Butko, Laura Schmalzl, Mark A. Williams

**Affiliations:** ^1^School of Psychology, University of East LondonLondon, UK; ^2^Perception in Action Research Centre, and ARC Centre of Excellence in Cognition and its Disorders, Department of Cognitive Science, Faculty of Human Sciences, Macquarie UniversitySydney, NSW, Australia; ^3^School of Psychology, and ARC Centre of Excellence in Cognition and its Disorders, University of Western AustraliaCrawley, WA, Australia; ^4^Department of Family and Preventive Medicine, University of California San DiegoLa Jolla, CA, USA

**Keywords:** face perception, body perception, object perception, prosopagnosia, MVPA, multivariate analysis, unfamiliar face, fMRI

## Abstract

The ability to identify faces is mediated by a network of cortical and subcortical brain regions in humans. It is still a matter of debate which regions represent the functional substrate of congenital prosopagnosia (CP), a condition characterized by a lifelong impairment in face recognition, and affecting around 2.5% of the general population. Here, we used functional Magnetic Resonance Imaging (fMRI) to measure neural responses to faces, objects, bodies, and body-parts in a group of seven CPs and ten healthy control participants. Using multi-voxel pattern analysis (MVPA) of the fMRI data we demonstrate that neural activity within the “core” (i.e., occipital face area and fusiform face area) and “extended” (i.e., anterior temporal cortex) face regions in CPs showed reduced discriminability between faces and objects. Reduced differentiation between faces and objects in CP was also seen in the right parahippocampal cortex. In contrast, discriminability between faces and bodies/body-parts and objects and bodies/body-parts across the ventral visual system was typical in CPs. In addition to MVPA analysis, we also ran traditional mass-univariate analysis, which failed to show any group differences in face and object discriminability. In sum, these findings demonstrate (i) face-object representations impairments in CP which encompass both the “core” and “extended” face regions, and (ii) superior power of MVPA in detecting group differences.

## Introduction

People are typically able to recognize hundreds of familiar faces with ease. Regions within the inferior occipital cortex (i.e., occipital face area, OFA), fusiform gyrus (i.e., fusiform face area, FFA), and anterior temporal lobe (AT) are part of a neural network that supports this extraordinary ability (Haxby et al., 2000; Ishai, 2008; Kanwisher, 2010). In particular, the OFA and the FFA are argued to represent “core” regions supporting the perception and recognition of visually presented faces, whereas the AT is considered an “extended” region, which mediates aspects of identity, name, and biographical information (Haxby et al., 2000; Kriegeskorte et al., 2007). Functional Magnetic Resonance Imaging (fMRI) studies have shown that these regions play a critical role in the recognition of facial identity. For instance, OFA and FFA fMRI activity is correlated with behavioral measures of face recognition ability (Yovel and Kanwisher, 2005; Kriegeskorte et al., 2007; Furl et al., 2011). In addition, brain injuries encompassing at least one of these regions often results in severe face recognition deficits (i.e., acquired prosopagnosia) (Barton, 2008; Rossion, 2008).

Face recognition difficulties are also apparent in approximately 2–3% of the general adult population with no reported brain injuries (Kennerknecht et al., 2006; Bowles et al., 2009; Wilmer et al., 2010). This specific difficulty in recognizing faces, in the context of otherwise intact sensory and intellectual functioning, is known as developmental or *congenital prosopagnosia* (CP) (McConachie, 1976; Duchaine, 2000; Behrmann and Avidan, 2005; Duchaine and Nakayama, 2006b; Schmalzl et al., 2008; Rivolta et al., 2010, 2012a). Some people with CP do not have difficulty differentiating between other similar objects (Behrmann et al., 2005; Wilson et al., 2010), whereas some people do (Duchaine et al., 2007; Lobmaier et al., 2010).

The neuro-functional correlates of CP are still far from clear. Two single case studies of CP reported atypical functioning of the FFA (Hadjikhani and De Gelder, 2002; Bentin et al., 2007). The FFA was also implicated in a study by Furl et al. (2011), who functionally localized ROIs (i.e., by contrasting faces—cars fMRI activity) and found weaker peak activity and a smaller number of fusiform gyrus face-voxels in a group of 15 CPs as compared to matched controls in these ROIs. However, there was no difference between CPs and controls when a whole brain analysis was conducted. Repetition suppression paradigms have typically indicated that both CPs and controls show a diminished fMRI signal to the repeated presentation of faces within the OFA and FFA (Avidan et al., 2005; Avidan and Behrmann, 2009; Furl et al., 2011). In contrast, other studies have not demonstrated atypical activity in core regions (i.e., the OFA or FFA) of CPs (Hasson et al., 2003; Avidan et al., 2005; Avidan and Behrmann, 2009). Typical face sensitive occipital and fusiform activity has also been demonstrated with magnetoencephalography (MEG) in a group of six CPs when considering source-reconstructed event-related fields (ERFs) activity (Rivolta et al., 2012b). Thus, previous fMRI and MEG studies suggest that posterior face activity may be necessary, but not sufficient, for normal face recognition (see Rossion, 2008 for similar arguments based on acquired prosopagnosia patients), and leaves open the possibility that regions outside the “core” OFA and FFA may play an important role in the behavioral face recognition difficulties underlying CP.

Support for the involvement of “extended” systems in face identity recognition comes from a recent fMRI study that showed that a group of seven CPs showed reduced AT activity for famous faces compared to controls, and also reduced AT functional connectivity with “core” face regions (Avidan et al., 2013). This study also showed relatively intact OFA and FFA activity, thus providing a functional dissociation between spared “core” face regions and impaired “extended” regions in CP. Aberrant functioning of the AT in CP is also in line with anatomical data showing AT volume reduction in CP (Behrmann et al., 2007) and reduced anatomical connectivity of the AT regions in CP (Thomas et al., 2009). This data, thus, supports proposals that CP is a disconnection syndrome where, due to anatomical and functional deficiencies, intact “core” face regions cannot pass their information to more anterior “extended” regions (Avidan et al., 2013; Rivolta et al., 2013).

Taken together, we see an inconsistent pattern across studies, with some showing OFA and FFA dysfunction, but others showing only AT abnormalities. While these differences may have been driven by the heterogeneity of CP itself (Schmalzl et al., 2008), they may also be the result of the power and sensitivity of the fMRI analysis approach adopted so far in CP literature. In particular, all previous fMRI studies investigating face processing skills in CP have used traditional mass univariate analysis. Recent evidence has, however, suggested that multivariate analysis of fMRI datasets MVPA provides a more sensitive analytical approach than traditional univariate analysis (Cox and Savoy, 2003; Haynes and Rees, 2006; Norman et al., 2006). In addition, univariate analyses may be less sensitive to AT regions activity (Mur et al., 2009), which is susceptible of signal distortion due to the ear canals and sinuses (Ojemann et al., 1997). Here, we use MVPA for the first time to investigate face processing activity in a group of seven CPs and 10 matched controls.

In addition to presenting faces and objects/scenes as visual stimuli (as in most previous neuroimaging CP investigations), in the current study we have also included body and body parts. In fact, bodies not only match faces for visual exposure and perceptual experience (Reed et al., 2012), but there is also evidence suggesting that body perception shares perceptual mechanisms (i.e., holistic processing) with faces (Reed et al., 2003; Willems et al., 2014), and that the processing of bodies can be impaired in CP (Righart and de Gelder, 2007; Van den Stock et al., 2008). Thus, participants were presented with visual stimuli from four different categories (faces, headless bodies, body parts, and objects) and their task was to press a button whenever a stimulus was repeated twice (i.e., one-back task).

## Methods and results

### Participants

Seven people with CP (4 Females, Mean age = 39.7, Range: 22–58, *SD* = 14.30) and 10 people who did not report face processing impairments (4 Females, Mean age = 33.6, Range: 27–55, *SD* = 9.55) completed the experiment. All participants reported normal or corrected to normal vision, no history of neurological or psychiatric conditions and all except one CP were right handed. All participants provided written consent after the experimental procedure was explained. The study received ethic approval from Macquarie University and it conforms to The Code of Ethics of the World Medical Association (Declaration of Helsinki), printed in the *British Medical Journal* (18th July 1964).

### Tasks used to confirm CP

All participants with CP were recruited through the online Australian Prosopagnosia Register (https://www.maccs.mq.edu.au/research/projects/prosopagnosia/register), where they registered because they were experiencing face recognition difficulties in everyday life. For detailed behavioral data of all CPs see Rivolta et al. (2012a). The CPs completed three tests of face identity recognition: (i) The MACCS Famous Face Test 2008 (MFFT-08), which measures the famous faces identification abilities (Palermo et al., 2011); (ii) The Cambridge Face Memory Test (CFMT, Duchaine and Nakayama, 2006a), which measures the memory for newly learned faces; and (iii) the Cambridge Face Perception Test (CFPT, Duchaine et al., 2007), which assesses face-matching abilities. A participant was considered CP if the performance on at least one of these three diagnostic tasks was at least 2 *SD* below the mean see Table 1 for age standardized z-scores calculated from the normative data in Bowles et al. (2009).

**Table 1:** **CPs' age and sex standardized z-scores on the MACCS Famous Face Test 2008 (MFFT-08), Cambridge Face Memory Task (CFMT), and Cambridge Face Perception Task (CFPT)**.

**CPs**	**Age**	**Sex**	**MFFT-08**	**CFMT**	**CFPT**
OJ	53	M	*−2.46*	*−2.72*	0.53
SD	57	M	*−3.1*	*−2.83*	−1.93
GN	47	F	*−4.05*	−1.81	−1.41
NN	24	F	*−4.5*	−1.93	−0.94
GE	22	M	*−2.04*	−1.89	−0.79
MG	33	F	*−3.49*	*−2.09*	*−2.86*
LL	41	F	*−2.43*	*−2.16*	*−2.95*

Scores falling more than 2 SD below the mean are displayed in italics.

Further tasks were administered to exclude that their face processing difficulties were consequence of low-level vision problems, general cognitive difficulties or impaired social functioning. All CPs showed normal contrast sensitivity as assessed by the *Functional Acuity Contrast Test* (FACT, Vision Sciences Research Corporation 2002) and normal color perception with the *Ishihara Test for Color Blindness* (Ishihara, 1925). Performance on the length, size, orientation and picture naming (long version) subtests of the *Birmingham Object Recognition Battery* (BORB) (Riddoch and Humphreys, 1993) confirmed that basic object recognition skills were intact. The *Raven Colored Progressive Matrices* (Raven et al., 1998) further indicated that the IQ of all participants with CP was within the normal range. None of the CPs scored within the autistic range on the *Autism-Spectrum Quotient* (AQ, Baron-Cohen et al., 2001). Thus, the everyday face recognition difficulties reported by the CPs are not due to low-level visual difficulties, low IQ, or impaired social functioning. All participants did not report any sign of anatomical brain alterations. Anatomical volumes (i.e., structural MRIs) have been routinely checked by an expert physician at S. Vincent's Hospital (Sydney).

### MRI data acquisition

Functional images were acquired with a 3-Tesla Philips scanner at St Vincent's Hospital (Sydney, New South Wales, Australia). At the beginning of the experimental session a high-resolution anatomical scan was acquired for each participant using a 3D-MPRAGE (magnetization prepared rapid gradient echo) sequence. Subsequently, high-resolution functional scans were obtained using an 8-channel head coil and a gradient echo planar imaging (EPI) sequence (114 time points per run; Inter-scan interval: 2 s, *TR* = 3000 ms, *TE* = 32 ms, voxel size = 1.4 × 1.4 × 2.0 mm; inter-slice gap: 20%). The 15 oblique axial slices were aligned approximately parallel to the anterior / posterior commissure line.

### fMRI experiment

#### Behavioral task: the one-back task

During the experiment participants were presented with visual stimuli belonging to four different categories: faces, headless bodies, individual body-parts (hands and feet) and objects. All stimuli were grayscale photographs and matched for brightness and contrast. The set of stimuli included a total of 240 images, 60 for each of the four stimulus categories (half of the “face” and “body” stimuli were females and half males). Stimuli covered approximately 4.1° of visual angle.

The presentation of stimuli during the fMRI acquisition was programmed with Presentation software (Neurobehavioral Systems, Albany, CA; http://www.neurobs.com/) and run on a 15-inch Macintosh Power Book with screen resolution set to 1280 × 854 pixels. Stimuli were back-projected via a projector onto a screen positioned 1.5 m behind the fMRI scanner, and participants viewed the screen through a mirror mounted on the head-coil and positioned at 10 cm distance from their head. An optic fiber button box was used to record the participants' responses.

Participants' brain activity was recorded in 8 functional runs with the duration of 336 s each. During each run, 114 functional scans (TRs) were acquired. The stimulus categories were presented in a blocked design with a total of 32 blocks of 16 s each. Each of the 32 blocks contained 16 stimuli of a specific category. Stimuli were presented in the center of the screen for 500 ms with a 500 ms inter-stimulus interval (ISI). The maintenance of attention to the stimuli was ensured by presenting participants with a standard “one-back” task. The task required pressing a button whenever a particular image was repeated consecutively (10% of the trials was a repeat). The order of blocks was counterbalanced across subjects. In addition, a fixation block (where a fixation cross was presented in the middle of the white screen) was presented at the beginning of each block and at the end of each fourth block (which corresponded to the end of the functional run).

#### One-back task performance

The one-back task was administered to ensure that participants were paying attention to the stimuli. Performance on the one-back task was analyzed by running a repeated-measures ANOVA with Group (controls, CPs) as a between-subject factor and Category (face, body, body part, object) as a within-subject factor. Performance on the one-back task did not differ between Controls (*M* = 0.771, s.e.m. = 0.185) and CPs (*M* = 0.722, s.e.m. = 0.221), *F*_(1, 15)_ = 2.9, *p* = 0.109. This was the case across all stimulus categories no main effect of Category [*F*_(3, 45)_ = 1.79, *p* = 0.163]; no Category by Group interaction [*F*_(3, 45)_ = 1.32, *p* = 0.277], which is not surprising given that the one-back task was relatively simple and could be completed by simply attending to only part of the image.

#### fMRI processing and multi-voxel pattern analysis (MVPA)

Preprocessing of the fMRI data was carried out using SPM8 (Wellcome Department of Imaging Neuroscience, London, UK; www.fil.ion.ucl.ac.uk). All EPI images were spatially realigned to the mean functional image and smoothed with a 4 mm full-width at half maximum (FWHM) kernel. The timecourse of each voxel was high-pass filtered with a cut off of 128 s.

Multi-voxel pattern analysis (MVPA) was used to discriminate patterns of activation pertaining to face, object, bodies, and body parts in each participant separately. These analyses used spatially realigned smoothed native space images which were additionally smoothed with a 4 mm (FWHM) kernel. First, for each participant, the multiple regression approach of SPM8 was used to estimate the response to each of face, body, body part, and fixation blocks in each of the 8 scanning acquisition runs, with additional regressors of no interest included to model the run means. Blocks were modeled using 16 s box car functions convolved with the canonical haemodynamic response function of SPM. This yielded 8 beta estimates for each of the face, object, body, and body part conditions (one for each run). Next, MVPA was used to estimate the pair-wise discriminability of these beta estimates using a roaming searchlight (Kriegeskorte et al., 2006). The approach identifies voxels where the pattern of activation in its local neighborhood can discriminate between conditions.

The analysis of face vs. object proceeded as follows. For each participant, the pattern of beta values from the 16 relevant images (8 faces and 8 objects) was extracted from a spherical ROI (radius, 10 mm) centered in turn on each voxel in the brain, yielding 16 multivoxel vectors. All the voxels in each sphere contributed to each vector, without feature selection. A linear support vector machine, LinearCSVMC (Chang and Lin, 2011), was trained to discriminate between the vectors pertaining to faces and those pertaining to objects. We used a leave-one-out 8-fold splitter: the classifier was trained using the data from 7 of the 8 runs and was subsequently tested on its accuracy at classifying the unseen data from the remaining run. This process was performed in 8 iterations, using all 8 possible combinations of train and test runs. The classification accuracies from the 8 iterations were then averaged to give a mean accuracy score for that sphere, which was assigned to the central voxel. This procedure was repeated for every voxel in the brain yielding whole-brain classification accuracy maps for each individual. This analysis was carried out using custom Matlab scripts wrapping the LIBSVM library (Chang and Lin, 2011). Finally, to combine data across individuals, the normalization parameters derived from normalizing the mean EPI image for each participant were used to normalize the classification accuracy maps. Accuracy maps for control and CP participants, separately, were entered into one-sample *t*-tests comparing group accuracy scores to chance (50%). The resulting whole brain statistical maps were then thresholded at *t* > 8.403, equivalent to *p* < 0.05 with Family Wise Error (FWE) correction in the control group analysis. This analysis reveals voxels where the local patterns of activation reliably discriminate between faces and objects across each group separately. To identify regions where face vs. object discrimination was significantly greater in controls relative to CPs, the accuracy maps were additionally entered into a two-sample *t*-test (control minus patient). The resulting whole brain statistical map was then thresholded to visualize clusters surviving cluster level correction for multiple comparisons at *p* < 0.05. The same procedure was carried out for the discrimination of faces vs. objects, faces vs. bodies, and faces vs. body-parts.

#### MVPA results

**Figure 1:**
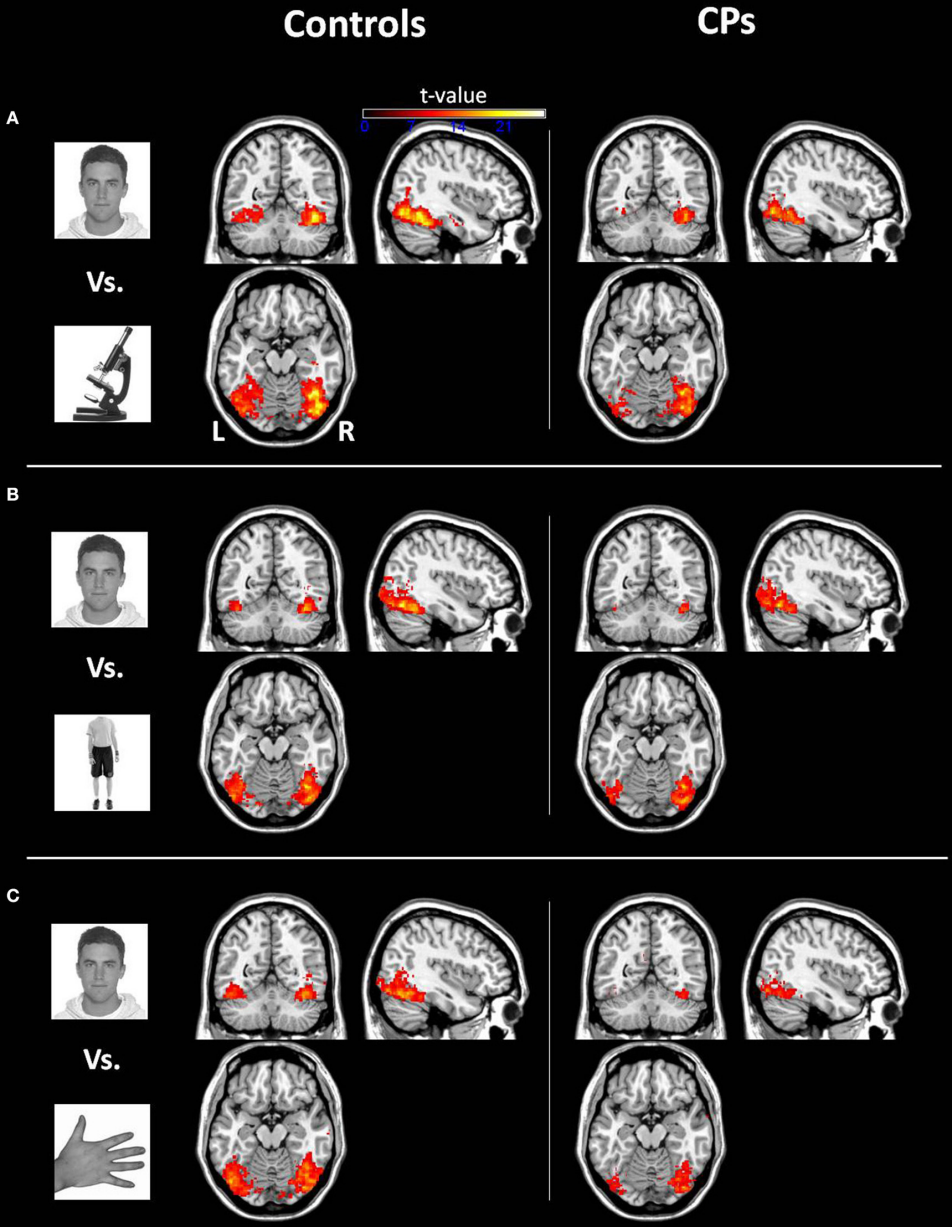
**Within-group analysis: Voxels where the local pattern of activation discriminates between (A) face vs. object, (B) face vs. body, and (C) face vs. body part (threshold: *t* > 8.40)**. Effects are shown for controls (left) and CPs (right).

**Figure 2:**
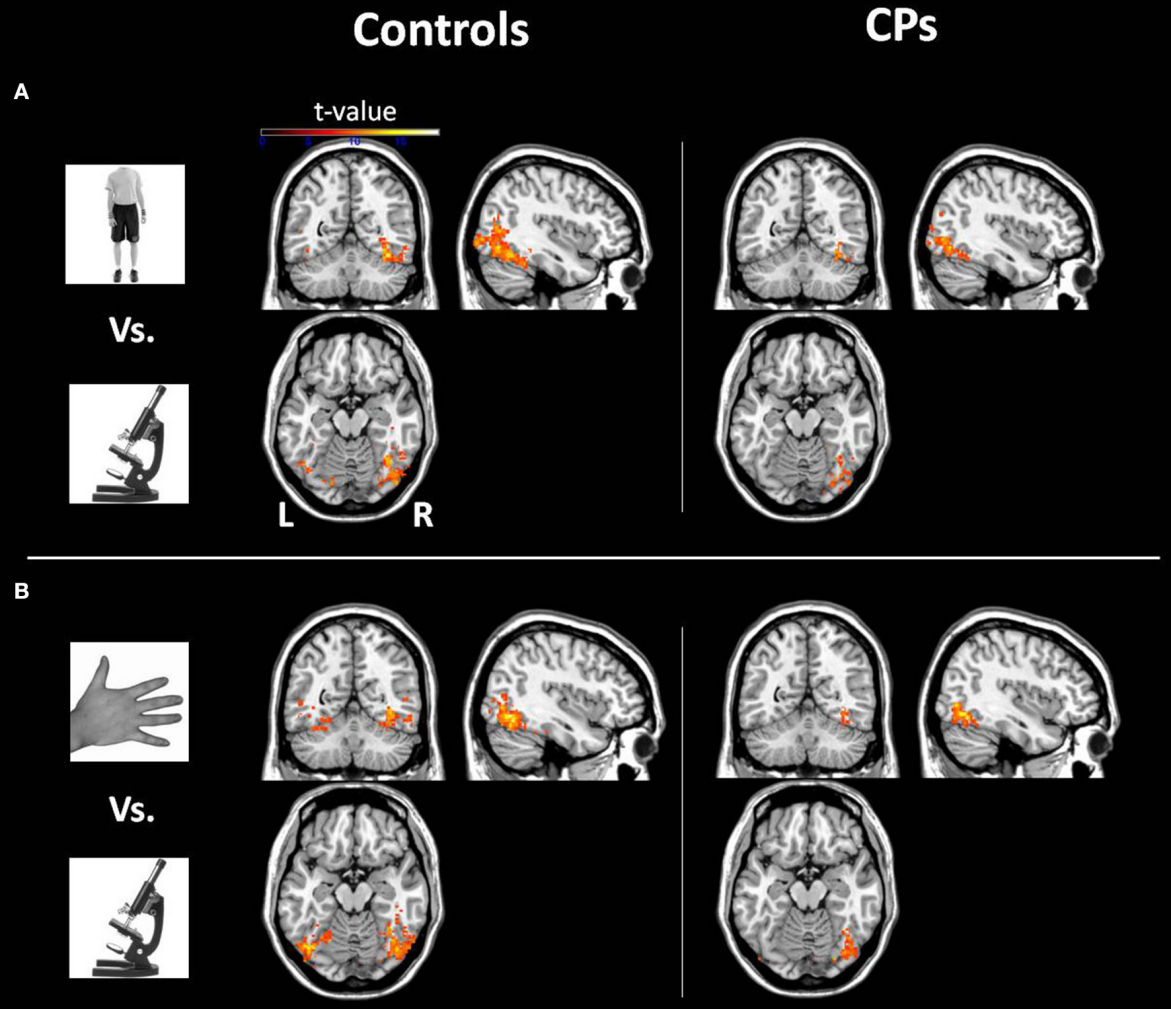
**Within-group analysis: Voxels where the local pattern of activation discriminates between (A) object vs. body and (B) object vs. body part (threshold: *t* > 8.40)**. Effects are shown for controls (left) and CPs (right).

***Within-group analyses: controls and CPs.*** Controls showed an above chance discrimination pattern between faces and objects over the fusiform gyri and inferior occipital gyri (see Figure 1 and Table 2). Controls also showed above chance discrimination between faces and bodies in the fusiform gyri, left middle occipital gyrus and lateral inferior occipital gyri (see Figure 1 and Table 2), and above chance discrimination between faces and body parts over fusiform gyri, left inferior temporal gyrus, lingual gyri, left superior occipital gyrus, right middle occipital gyrus, and lateral inferior occipital gyri (see Figure 1 and Table 2). Controls' pattern of activity could above chance discriminate between object and bodies over the left inferior occipital gyrus, right middle occipital gyrus, and right fusiform gyrus (see Figure 2 and Table 2). Finally, controls showed an above chance discrimination pattern between object and body parts over the inferior occipital gyrus (bilateral), fusiform gyrus (bilateral), right lingual gyrus, left inferior temporal gyrus, and right middle temporal gyrus (see Figure 2 and Table 2).

**Table 2:** **Anatomical regions (Label), MNI coordinates (x, y, z), *z*-values (*z*-value), Brodmann areas (BA), clusters sizes (KE), and sides (L, Left; R, Right) of the within- (Controls and CPs) and between- (Controls vs. CPs) group effects**.

	**Label**	***x***	***y***	***z***	***z*-value**	***BA***	***KE***	**Side**
**FACE Vs. OBJECT**								
**Controls**								
Cluster 1	Fusiform gyrus	40	−56	−16	7.54	37	786	R
	Inferior occipital gyrus	44	−70	−12	7.39	19	/	R
Cluster 2	Fusiform gyrus	−42	−52	−24	6.96	37	23	L
Cluster 3	Fusiform gyrus	−48	−64	−22	6.92	37	190	L
	Inferior occipital gyrus	−44	−74	−4	5.79	19	/	L
**CPs**								
Cluster 1	Fusiform gyrus	32	−60	−18	7.27	37	302	R
Cluster 2	Inferior occipital gyrus	−42	−76	−12	6.55	19	32	L
	Middle occipital gyrus	−44	−75	0	6.55	18	/	L
Cluster 3	Fusiform gyrus	32	−44	−22	6.38	37	7	R
**Controls vs. CPs**								
Cluster 1	Inferior occipital gyrus	48	−76	−18	4.79	18	62	R
	Inferior temporal gyrus	48	−68	−8	3.29	20	/	R
Cluster 2	Fusiform gyrus	−48	−68	−20	4.50	19	/	L
Cluster 3	Parahippocampal gyrus	34	−14	−26	4.39	20	44	R
	Fusiform gyrus	42	−14	−28	3.66	20	/	R
	Inferior temporal gyrus	40	−6	−28	3.3	20	/	R
Cluster 4	Fusiform gyrus	40	−56	−16	4.21	20	33	R
**FACE Vs. BODY**								
**Controls**								
Cluster 1	Fusiform gyrus	40	−68	−18	7.38	37	316	R
Cluster 2	Inferior occipital gyrus	−30	−94	−6	6.9	19	13	L
Cluster 3	Inferior occipital gyrus	−46	−80	−6	6.86	19	118	L
	Fusiform gyrus	−44	−75	−18	5.83	37	/	L
	Middle occipital gyrus	−42	−84	0	5.51	18	/	L
Cluster 4	Fusiform gyrus	−40	−70	−18	6.64	19	26	L
Cluster 5	Inferior occipital gyrus	34	−96	−2	6.45	18	12	R
Cluster 6	Fusiform gyrus	−40	−56	−20	6.43	37	9	L
**CPs**								
Cluster 1	Fusiform gyrus	36	−64	−14	7.12	37	51	R
Cluster 2	Lingual Gyrus	40	−82	−16	6.93	19	101	R
	Inferior occipital gyrus	35	−85	−8	6.52	19	/	R
	Fusiform gyrus	28	−75	−14	6.5	37	/	R
Cluster 3	Inferior occipital gyrus	−46	−80	−6	6.9	19	54	L
	Middle occipital gyrus	−42	−84	0	5.58	18	/	L
Cluster 4	Fusiform gyrus	36	−58	−24	6.62	37	14	R
Cluster 5	Middle occipital gyrus	−32	−92	−6	6.55	18	8	L
Cluster 6	Inferior occipital gyrus	34	−96	−2	6.4	19	6	R
Cluster 7	Inferior occipital gyrus	50	−80	−2	6.34	19	7	R
**FACE Vs. BODY PART**								
**Controls**								
Cluster 1	Inferior occipital gyrus	−44	−76	−10	7.3	19	300	L
	Fusiform gyrus	−45	−72	−20	7.01	37	/	L
	Inferior temporal gyrus	−48	−55	−5	6.94	20	/	L
Cluster 2	Fusiform gyrus	38	−66	−20	7	37	265	R
Cluster 3	Inferior occipital gyrus	−34	−84	−10	6.98	19	16	L
Cluster 4	Lingual Gyrus	12	−90	−8	6.86	19	17	R
Cluster 5	Lingual Gyrus	−30	−84	−12	6.62	19	12	L
Cluster 6	Middle occipital gyrus	−40	−90	−4	6.73	18	7	L
Cluster 7	Fusiform gyrus	30	−86	−14	6.5	37	12	R
Cluster 8	Superior occipital gyrus	−14	−98	10	6.44	17	10	L
Cluster 9	Middle occipital gyrus	30	−94	2	6.4	18	10	R
**CPs**								
Cluster 1	Inferior occipital gyrus	−48	−82	−10	6.93	19	32	L
Cluster 2	Lingual Gyrus	38	−80	−16	6.75	19	25	R
**OBJECT VS. BODY**								
**Controls**								
Cluster 1	Inferior occipital gyrus	−44	−72	−2	6.88	19	28	L
	Middle occipital gyrus	44	−74	6	6.64	18	/	R
Cluster 2	Fusiform gyrus	38	−56	−18	6.57	37	9	R
**CPs**								
Cluster 1	Inferior occipital gyrus	44	−72	−4	6.57	19	6	R
**OBJECT VS. BODY PART**								
**Controls**								
Cluster 1	Inferior occipital gyrus	38	−66	−10	7.28	19	1181	R
	Fusiform gyrus	36	−58	−8	6.7	37	/	R
	Fusiform gyrus	35	−54	−15	6.35	37	/	R
Cluster 2	Fusiform gyrus	−42	−88	−12	6.54	37	1076	L
	Inferior temporal gyrus	−52	−58	−5	6.45	20	/	L
	Fusiform gyrus	−42	−70	−16	6.38	37	/	L
Cluster 3	Lingual gyrus	22	−78	−8	5.88	19	16	R
Cluster 4	Inferior occipital gyrus	26	−100	0	5.85	19	28	R
Cluster 5	Fusiform gyrus	−32	−34	−26	5.78	37	12	L
Cluster 6	Inferior occipital gyrus	−20	−98	−10	5.65	19	7	L
Cluster 7	Inferior temporal gyrus	−54	−60	12	5.51	20	23	L
Cluster 8	Inferior occipital gyrus	−40	−68	−8	5.47	19	7	L
Cluster 9	Middle temporal gyrus	36	−68	10	5.47	37	8	R
**CPs**								
Cluster 1	Inferior occipital gyrus	38	−66	−10	6.98	19	537	R
	Inferior occipital gyrus	42	−74	−2	5.35	19	/	R
	Fusiform gyrus	35	−54	−15	5.05	37	/	R
Cluster 2	Fusiform gyrus	−42	−88	−12	6.71	37	271	L
	Middle occipital gyrus	−44	−85	−4	5.91	18	/	L
	Middle occipital gyrus	−50	−75	2	5.78	18	/	L
Cluster 3	Inferior temporal gyrus	46	−52	−24	5.82	20	7	R
Cluster 4	Lingual gyrus	22	−78	−8	5.55	19	17	R

Contrasts reported: face vs. object, face vs. body, face vs. body-part object vs. body and object vs. body part.

CPs' MVPA activity over the right fusiform gyrus, left middle occipital gyrus, and left inferior occipital gyrus could discriminate between faces and objects at levels above-chance (see Figure 1 and Table 2). CPs also showed above chance discrimination between faces and bodies in the right fusiform gyrus, right lingual gyrus, left middle occipital gyrus, and inferior occipital gyri (see Figure 1 and Table 2), and above chance discrimination between faces and body parts over the left inferior occipital gyrus and right lingual gyrus (see Figure 1 and Table 2). CPs' pattern of fMRI activity could discriminate between objects and bodies over the right inferior occipital gyrus. Finally, CPs showed an above chance discrimination pattern between the inferior occipital gyrus (bilateral), fusiform gyrus (bilateral), right lingual gyrus, left inferior temporal gyrus, and right middle temporal gyrus (see Figure 2 and Table 2).

**Figure 3:**
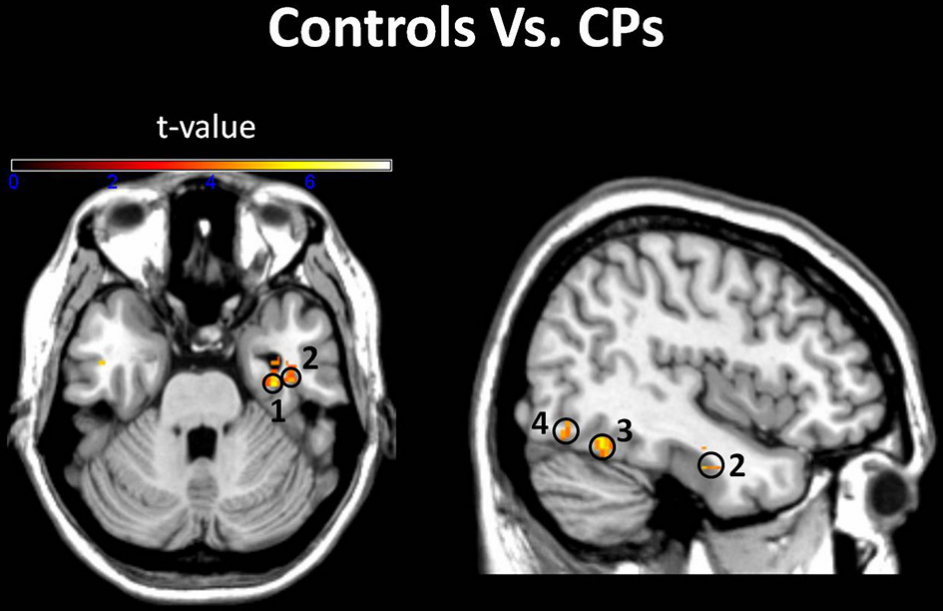
**Groups comparison**. Voxels where the local pattern of activity discriminated faces from objects more strongly in controls than in CPs: (1) right parahippocampal gyrus [34 −14 −26]; (2) right inferior temporal gyrus [40 −6 −28]; (3) right fusiform Gyrus [40 −56 −16]; (4) right inferior occipital gyrus [48 −76 −18] (threshold: *t* > 3.73).

***Between-group analyses: controls vs. CPs.*** The between-groups comparison indicated stronger face-object discrimination in controls than in CP. This group difference was evident in the fusiform gyri, right inferior occipital gyrus, right inferior temporal gyrus, and right parahippocampal gyrus (Figure 3 and Table 2). The two groups' MVPA activity did not differ when discriminating faces vs. bodies, faces vs. body parts, objects vs. bodies, and objects vs. body parts.

#### fMRI mass-univariate analysis

To compare our MVPA results to standard fMRI univariate findings, we performed a group level whole-brain mass-univariate statistic as implemented in SPM. To define face-sensitive regions, we compared faces vs. objects. Processing of all EPI images follows standard SPM procedure. All EPI images were normalized to T1-weightened MNI structural template and smoothed with an 4 mm Gaussian filter. As for the multivariate analysis, the multiple regression approach of SPM8 was used to estimate the response to each block in each of the 8 scanning acquisition runs, for each participant, with additional regressors of no interest included to model the run means. Blocks were modeled using 16 s box car functions convolved with the canonical haemodynamic response function of SPM. This yielded 8 beta estimates for each condition; one for each run. To find face discriminating region in each group (controls, patients), a one-sample *t*-test was performed for each group separately using face minus object contrasts as reference images. The resulting map was thresholded at *t* > 8.403, equivalent to *p* < 0.05 with FWE correction. A between groups (controls minus patients) comparisons using two-sample independent *t*-test with unequal variance was performed with face minus object contrast images. The resulting whole brain statistical map was then thresholded to visualize clusters surviving cluster level correction for multiple comparisons at *p* < 0.05. In addition, face selective regions were also investigated in each subject separately (i.e., *single-subject analysis*) by contrasting the BOLD signal associated with presentation of faces compared to objects at the single subject level (*p* < 0.05 FWE).

#### fMRI results: mass univariate analysis

***Within-group analyses: Controls and CPs.*** At the group level, using the same threshold that was used in the MVPA analysis (*t* > 8.403), we could not find any statistically significant fMRI activity (face-sensitive activity could not be found even with a more permissive threshold of *p* < 0.001 uncorrected). Since this lack of group activity could potentially be due to the between-subject variability in the location of face-sensitive regions, we additionally performed single-subject analyses, where we compared face vs. object activity. Results, in line with previous studies (e.g., Avidan et al., 2013), indicated that all controls show “core” face activity in the right OFA and FFA. Five out of seven CPs also showed OFA and four CPs showed FFA (Table 3).

**Table 3:** **Core face regions (i.e., OFA, FFA, STS) activity in the right (R) and left (L) hemisphere for both controls and CPs**.

	**FFA-R**	**OFA-R**	**STS-R**	**FFA-L**	**OFA-L**	**STS-L**
**CONTROLS**						
S01	x	x	x	x	x	
S02	x	x	x	x		
S03	x	x	x			
S04	x	x	x	x	x	
S05	x	x	x			
S06	x	x	x	x	x	x
S07	x	x	x		x	
S08	x	x	x	x	x	
S09	x	x				
S10	x	x				
**CPs**						
OJ						
GN			x			
LL	x	x	x		x	
NN	x	x	x	x	x	
MG	x	x	x	x		x
OJ		x			x	
SD	x	x			x	

“x” indicates the presence of a particular face region in a subject, whereas a blank space indicates its absence (activity thresholded at p < 0.05 FWE).

***Between-group analyses: controls vs. CPs.*** The group comparison did not show any statistically significant difference between controls and CPs. Thus, as predicted, mass-univariate analysis is not as sensitive as MVPA in detecting group differences. Given the small number of single-subject localized face-sensitive regions in CPs (see Table 3), we did not run any statistical analysis to compare the two groups.

## Discussion

We investigated the neural characteristics of CP by examining the pattern of activity to faces, objects, headless bodies, and body parts using MVPA. We found that the pattern of fMRI activity within both the “core” and “extended” face regions showed reduced sensitivity discriminating faces and objects in a group of seven CPs as compared to a group of control participants. For the first time, we also report that this pattern poor discrimination between faces and objects in CPs is also evident in the right parahippocampal gyrus. The two groups did not show any difference in face-body, face-body part, object-body, and object-body part discriminations. Given that mass-univariate results failed to report any group difference, we can also conclude that MVPA represents a more sensitive approach than traditional univariate statistics in detecting group differences (Norman et al., 2006). Note that since only the face-object contrast showed group differences and that the univariate analysis failed to report differences between controls and CPs, we exclude that group differences can be explained in term of general activity differences.

We acknowledge that face-sensitive regions (e.g., OFA and FFA) are traditionally defined using traditional mass-univariate analysis (Kanwisher et al., 1997). In the current study, due to the lack of face-sensitive (i.e., univariate) regions, we could not localize, at a *group level*, OFA, FFA and AT. In addition, we could not ascertain whether MVPA-defined face-object discriminant regions (Figures 2, 3) include or not OFA, FFA, and AT. However, in order to compare the current study to previous findings in CP, we label the MVPA activities in the lateral occipital, fusiform and AT cortex as, respectively, OFA, FFA, and AT (Figures 1, 2).

Results showed that, in controls, OFA and the FFA activity could discriminate between face and non-face (i.e., objects, bodies, body parts) stimuli above-chance (Figure 1). This result is in line with previous human neuroimaging (Pitcher et al., 2009), lesion (Barton, 2008), and animal (Tsao et al., 2008a) studies indicating the critical role of the ventral visual system for face, body, and object processing (see Yovel and Freiwald, 2013 for a review). Despite the finding that occipito-temporal regions in people with CP could be used to discriminate face vs. non-face stimuli (Figure 1), the crucial direct comparison between CPs and control participants demonstrated reduced face-object discriminatory pattern in CP, which was evident in the right OFA, bilateral FFA, right AT, and right parahippocampal gyrus (Figure 2). The finding of OFA and FFA functional aberrations is in line with previous single case studies showing reduced (or absent) posterior face activity in CP (Hadjikhani and De Gelder, 2002; Bentin et al., 2007). However, this result is in disagreement with recent studies in groups of CP which show typical “core” albeit impaired “extended” face regions (Avidan and Behrmann, 2009; Avidan et al., 2013), and points toward the better sensitivity of MVPA with respect to univariate analysis of group fMRI data. In addition, “core” face-region aberrations demonstrate the crucial involvement of early face regions in CP, thus potentially positing against a “disconnection syndrome,” which characterizes CP as the result of the functional isolation between (relatively spared) posterior face regions and (impaired) anterior face nodes (Avidan et al., 2013; Rivolta et al., 2013).

In agreement with Avidan et al. (2013), we reported atypical AT face-sensitive activity in CP. This finding further suggests the pivotal role of AT for typical face processing (Williams et al., 2006). However, in contrast to Avidan's et al. (2013), we also showed AT face-object group differences for unfamiliar, and not just famous, faces. Human (Rajimehr et al., 2009) and monkey (Tsao et al., 2008b) studies suggested that the AT patches respond to face stimuli in general, but are particularly sensitive to face identity. Given that the current study did not adopt familiar/famous faces and did not involve any identity or learning process, our finding of diminished unfamiliar-face vs. object discrimination in CP further demonstrates the sensitivity of MVPA analysis for the decoding of atypical neurophysiological properties of the human face recognition system.

A core face region that did not show MVPA face-object discriminant activity in either CPs or controls was the superior temporal sulcus (STS). The STS has been previously implicated in changeable aspects of face processing (Hoffman and Haxby, 2000; Puce and Perrett, 2003), facial emotions expression (Said et al., 2010) and facial dynamics (Schultz et al., 2013) (see Haxby et al., 2000 for a review). Given that we used static stimuli that did not show facial expressions, it is likely that that our experimental setting was not the most appropriate for engaging STS activity.

Overall, these results demonstrate for the first time with MVPA that both “core” and “extended” face regions show abnormal pattern of fMRI activity in CP. Thus, aberrant activity in a network including occipital and temporal regions mediates atypical face processing skills in CP. It is important to note, however, that since the MVPA analysis adopted only tests the for neural discrimination accuracy between category pairs (i.e., face vs. object), we cannot claim that the CP reduced face-object discrimination is truly face-specific. In theory, the CP aberrant discrimination pattern could have been equally driven by object or face processing. The lack of an object-body and object-body part group difference seems to exclude an object-specific coding problem. However, in the same fashion, the lack of face-body and face-body part group differences seems to rule out a face-specific problem in CP. Given the nature of the condition, which is often characterized by a disproportionate deficit in face processing (Duchaine and Nakayama, 2005), and given that the group differences appears in brain areas strongly implicated in face (Haxby et al., 2000; Avidan et al., 2013), rather than object (Kanwisher, 2010) processing, it seems however plausible to suggest that the group difference depicts a “face-driven” MVPA accuracy reduction in CP.

A finding never reported before in CP neuroimaging literature is the reduced face-object discrimination in the right parahippocampal gyrus. Given that the parahippocampal gyrus is a region strongly implicated in memory processing (Davachi et al., 2003) and involved in unfamiliar (Rivolta et al., 2014) and familiar (Leveroni et al., 2000) face perception, our results point toward a potential anatomical locus of face-object processing problems in CP. We note that the 1-back task did not tax memory, and CPs and controls did not differ in their performance on this task. It is, thus, possible that reduced face-object discrimination in the parahippocampal gyrus may reflect poor face memory in CP, as highlighted by their poor performance on the CFMT (see Table 1). Future studies which adopt tasks specifically tapping memorial aspects of face processing may clarify why reduced sensitivity was seen in this area.

Our finding of face-body, face-body part, object-body, and object-body part representations within the occipital and fusiform cortices (both in controls and CPs) are consistent with previous studies (Bar et al., 2006; Peelen and Downing, 2007) highlighting the importance of posterior ventral regions for body and object processing. The absence of group differences for face vs. body/body-parts activity albeit in agreement with previous behavioral studies suggesting typical body processing in CP (Duchaine et al., 2006), disagrees with previous EEG (Righart and de Gelder, 2007) and fMRI (Van den Stock et al., 2008) evidence reporting neurophysiological group differences, thus highlighting the need for future investigations.

## Conclusions

The current study demonstrates that face-object discriminatory abilities in the lateral occipital cortex, fusiform gyrus, AT cortex and parahippocampal gyrus are compromised in people with CP. Although our analysis cannot directly posit for a “face-driven” coding problem in CP, the clinical features of the condition and the localization of the group differences in well known “core” and “extended” face regions seems to posit for a pivotal contribution of CP face processing deficits for the neural pattern observed. Thus, both core- and extended- face networks appear to reflect the behavioral abnormality congenital prosopagnosics experience in everyday life and elucidates a neural marker of CP. Future studies should further investigate the face-specificity issue by, for instance, testing the neural representation of multiple exemplars of individual faces and objects.

### Conflict of interest statement

The authors declare that the research was conducted in the absence of any commercial or financial relationships that could be construed as a potential conflict of interest.
